# Amplification, purification, and lyophilization of mycobacteriophages for therapeutic use

**DOI:** 10.1128/spectrum.02277-25

**Published:** 2025-11-03

**Authors:** Maggie D. Viland, Lawrence Abad, Deborah Jacobs-Sera, Krista G. Freeman, Daniel A. Russell, Rebecca A. Garlena, Emily Mowry, Shengwei Yu, Michael J. Lauer, Reinhard Vehring, Carlos Guerrero-Bustamante, Graham F. Hatfull

**Affiliations:** 1 Department of Biological Sciences, University of Pittsburgh 171653, Pittsburgh, Pennsylvania, USA; 2 Department of Physics, Case Western Reserve University, Cleveland, Ohio, USA; 3 Department of Mechanical Engineering, University of Alberta 388644, Edmonton, Alberta, Canada; University of California San Diego, La Jolla, California, USA

**Keywords:** bacteriophages, *Mycobacterium*, phage preparation

## Abstract

Infections with nontuberculous mycobacterium (NTM) strains are frequently nonresponsive to antibiotic regimens, even after multiple drugs are used for extensive periods of months or years. Therapy with bacteriophages specific for NTM clinical isolates shows considerable promise in compassionate-use single-patient cases but, like antibiotics, must be administered over long periods of time and typically intravenously. It is thus important that the phage preparations are highly purified and stable to avoid adverse reactions or decay of viability. Here, we describe methods for purification of mycobacteriophages including CsCl equilibrium gradient ultracentrifugation, desalting by either dialysis or column chromatography, and stabilization for storage by lyophilization. These phage preparations have low levels of contaminating *Mycobacterium smegmatis* host proteins and host genomic DNA, and the lack of host DNA strongly indicates that these phages are not competent for generalized transduction. Desalting by column chromatography is both rapid and efficient for removal of CsCl after equilibrium density ultracentrifugation, and the preparations maintain viability well in lyophilization using trehalose as an excipient. Once lyophilized, the phage preparations typically maintain viability over extended periods of time, although once reconstituted at working dose concentrations and distributed into syringes, they experience about a 10-fold loss in viability over a 2-week period.

**IMPORTANCE:**

Bacteriophages specific for *Mycobacterium* hosts show promise as potential therapeutic agents for controlling nontuberculosis *Mycobacterium* infections. Phage administration for compassionate-use cases frequently involves intravenous twice-daily doses, but over a period of many months or years; the biosafety profile is, therefore, of substantial importance. Here, we describe the detailed methods we have used to grow mycobacteriophages to high titers, to concentrate and purify them so that they are devoid of major contaminants, and methods for stable long-term storage.

## INTRODUCTION

Bacteriophages have been considered potentially promising therapeutics for bacterial infections for over 100 years (1), but have received more recent attention in part because of the impending threat of antibiotic-resistant microbes (2, 3). Nontuberculous mycobacterium (NTM) infections, especially those of *Mycobacterium abscessus*, are challenging to treat clinically as many strains are nonresponsive to antibiotics due to both intrinsic and acquired resistance (4, 5). Experimental applications of *Mycobacterium* bacteriophages for treating NTM infections on a compassionate-use basis have shown considerable promise (6–9), although the limited phage repertoire and great strain variation in phage susceptibilities limit broader usage (10–12).

A large collection of mycobacteriophages have been isolated on *Mycobacterium smegmatis,* and over 2,600 of these have been sequenced and annotated, providing a high-resolution view of their genome diversity (13). Because relatively few phages have been isolated directly on any strain of *M. abscessus*, a small subset of *M. smegmatis* phages that also infect at least some strains of *M. abscessus* have shown the greatest clinical promise (12). However, many of these phages are temperate and thus need to be engineered to be obligatorily lytic and thus therapeutically suitable (6, 14). The advantage of using *M. smegmatis* phages is that they can be propagated using a well-behaved and predictable nonpathogenic host (*M. smegmatis*), although for therapeutic use, high-titer lysates still need to be concentrated, demonstrated to be sterile, endotoxin-free, and stable both prior to and during shipping and storage.

Phage amplification, purification, and storage are key parameters in all phage preparations for therapeutic use. Amplification is typically either in liquid growth or on solid medium, although several different purification methods are in common usage (15, 16). The cleanliness of the preparations is of particular concern when used for intravenous delivery because although phage particles themselves appear to present little biohazard (and there are an estimated 10^16^ particles naturally present in the human body) (17), contaminants in the preparation can present toxicity risks. For phages propagated on gram-negative bacterial hosts, lipopolysaccharide contaminants as endotoxins present a substantial risk (18), although this is not a concern for mycobacteriophage preparations, provided the bacterial cultures are not contaminated. Mycobacteriophages used for compassionate-use therapies to date have typically been purified through two rounds of equilibrium density CsCl ultracentrifugation. However, the CsCl must then be removed, and although dialysis can do this efficiently, some phages lose viability in the process (6, 7, 12). Lyophilization has long been used to prepare phages for storage (19) and has been recently described in phage used for therapy (20, 21). Phages processed by lyophilization need to be protected against stresses from freezing and desiccation. Various excipients that function as lyoprotectants or bulking agents have been used in combination with different processing conditions for lyophilization, all of which can impact phage stability (22, 23). There is little information on lyophilization of mycobacteriophages, although the recovery of part of a small collection of lyophilized mycobacteriophages following approximately 50 years of lyophilized storage has been reported (24). However, liquid preparations of mycobacteriophages, even at high titer (10^11^ PFU/mL), show varying stability in both storage and shipping, and alternative storage conditions are warranted.

Here, we describe methods used for the amplification, purification, and storage of mycobacteriophages for therapeutic use. Following purification in CsCl equilibrium density centrifugation, CsCl can be efficiently and rapidly removed by chromatography, and the phage particles can be lyophilized in ready-to-use aliquots with long-term storage and ambient-temperature shipping without substantial losses in viability.

## RESULTS

### Phage amplification and purification

**Fig 1:**
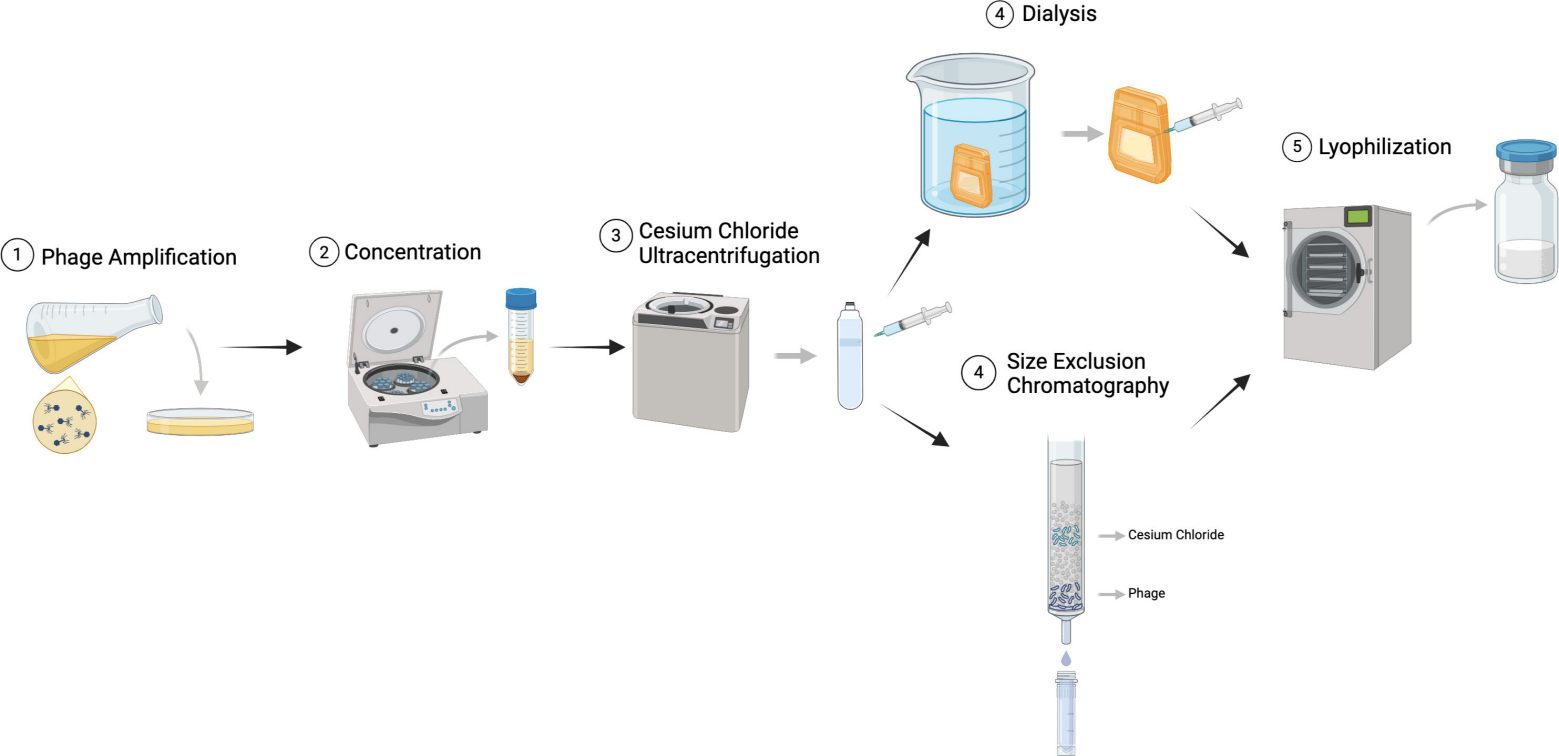
Scheme for mycobacteriophage amplification, purification, and lyophilization. Amplification of mycobacteriophages was done on the solid medium, the phage harvested, concentrated, and purified through two rounds of equilibrium density CsCl ultracentrifugation. The CsCl was then removed either by multiple cycles of dialysis or by column chromatography. Phage preparations were then aliquoted to glass vials and lyophilized. Created with BioRender.com.

The general scheme for phage amplification, purification, and storage is shown in Fig. 1, and the phages used are shown in Table 1. Phage amplifications were routinely performed using solid media, as in our experience with mycobacteriophages, higher titers are achieved than by amplification in the liquid medium. All of the phages reported for therapeutic use were propagated on *M. smegmatis*, although other *Mycobacterium* hosts could be used with appropriate adaptations. Typically, phage lysates harvested from the solid medium ranged from 10^10^ to 10^12^ PFU/mL, and using 40 150 mm diameter Petri dishes yielded approximately 200mL of the lysate. The phage particles were pelleted by high-speed centrifugation, resuspended in phage buffer, and subjected to two rounds of equilibrium density ultracentrifugation in CsCl. Precipitation with polyethylene glycol/sodium chloride can also be used for phage concentration, although in our experience, it can give inconsistent yields. Most phage preparations stored in the CsCl solution were quite stable when stored at 4°C over long periods. Eleven different preparations of six different phages stored from 2 to 30 months in CsCl showed an average percent loss of 15% per month of storage, ranging from 4.6% to 30.9% loss per month.

**TABLE 1:** Phages used in this study

Phage^*a*^	Cluster^*b*^	Morphotype	Genome (bp)^*c*^	Packaging^*d*^
Bxb1	A1	Siphophage	50,550	cos
Muddy	AB	Siphophage	48,229	cos
BPs (phKSW1)	G1	Siphophage	41,901	cos
Patience	U	Siphophage	70,506	cos
Fionnbharth	K4	Siphophage	58,075	cos
Dandelion	C1	Myophage	157,568	pac
CrimD (phSY1)	K1	Siphophage	59,798	cos

^*a*^ Phage names are shown, with some derivatives shown in parentheses.

^*b*^ Cluster or subcluster designations are shown.

^*c*^ Genome lengths are show for wild-type phages.

^*d*^ Packaging systems used by these phages.

### Determination of phage titer and purity

A prior investigation of mycobacteriophage purification using proteomic analysis with the phage Patience showed that, following one cycle of equilibrium density centrifugation in CsCl, the phage was highly enriched but still contained substantial components of both *M. smegmatis* host proteins and highly expressed phage nonstructural proteins (25). However, a second cycle of CsCl gradient purification substantially lowered the prevalence of these contaminating proteins (25). To evaluate the relative purity of therapeutically useful phages using the scheme shown in Fig. 1, we first used LC-MS/MS to determine the proportions of *M. smegmatis* proteins present (Table 2). Following two cycles of CsCl equilibrium density centrifugation, the proportion of *M. smegmatis* protein of spectra for all samples was low, less than 1% of the total spectra. Second, whole-genome sequencing was performed of several different phage preparations following two cycles of CsCl purification, mapping sequencing reads to the purified phage and to the *M. smegmatis* host genome (Table 3). For each sample, the number of total reads mapping to the host genome was very low (<0.001%) or none were detected (Table 3). These analyses indicate that phages prepared in this way have only low levels of contamination of nonphage components.

**TABLE 2:** Proteomic analysis of phage preparations

Phage	No. of spectra w/ no hits^*a*^	No. of phage spectra^*b*^	No. of host spectra^*b*^	% host spectra
Bxb1	175	5,916	7	0.12
Muddy	432	9,178	7	0.08
Patience	265	5,404	0	0.00
BPs	957	11,468	12	0.10
phKSW1	825	12,463	110	0.87
Fionnbharth	1,039	11,452	85	0.74

^*a*^ Spectra for LC-MS-MS with no hits to the peptide database.

^*b*^ Spectra for LC-MS-MS with hits to phage proteins or *M. smegmatis* proteins.

**TABLE 3:** Phage genome sequences^*d*^

Phage	Total no. of reads^*a*^	No. of phage reads^*b*^	Phage coverage^*c*^	No. of host reads^*d*^
Muddy	388,032	380,782	1,828	1
Muddy_HRM^N0052^	1,899,712	1,899,659	5,805	2
phSY1	1,562,746	1,562,718	3,761	0
Muddy_HRM^N0052^	1,678,082	1,678,010	4,952	0
phKSW1	7,603,876	7,603,349	31,584	63
BPsΔ*33*HTH_HRM^GD03^	5,958,404	5,957,977	24,530	2
Dandelion	833,652	826,001	1,174	196

^*a*^ Sequence reads were obtained either by Illumina-based sequencing on a MiSeq, NextSeq, or NovaSeq instrument.

^*b*^ Number of phage reads mapping to the phage.

^*c*^ Average number of times each base pair is represented.

^*d*^ Number of phage reads mapping to *M. smegmatis*.

We also sequenced a similarly purified phage “Dandelion” which groups in Cluster C, and some of these phages have been shown to be capable of generalized transduction (26, 27). To ensure that read mapping was accurate, reads mapped to the host genome were manually inspected. A total of 0.02% reads were mapped to the *M. smegmatis* genome, which plausibly arise from generalized transducing particles. Because so few reads mapped to the host genome in the preparations of Muddy or its derivatives or phSY1 (a derivative of CrimD), it seems unlikely that they mediate generalized transduction at any detectable level.

### Methods for CsCl removal

Following purification of phage particles by equilibrium density centrifugation, it is necessary to remove the CsCl. One well-established method is extensive dialysis (6), and we have typically performed this using Slide-A-Lyzer cassettes (Thermo Fisher, 66380), and dialyzing approximately 1 mL of phage solution against 1 L buffer (Ringer’s solution) using four buffer changes, over a total of about 44 hours. We have previously reported that this lowers the CsCl amount to below 190 parts per billion (6). We have also tested the residual CsCl concentrations of two additional phage preparations after dialysis, which were 40 and 132 ppb. There is relatively sparse information on the toxicity of nonradioisotopic Cs, although it has been used at relatively high doses in cancer treatments (several grams per day), and lower doses are considered generally safe (28). Inclusion of CsCl at 190 ppb is equivalent to 0.00019 mg in 1 mL, six orders of magnitude lower than that used in cancer therapies. However, we have observed that extensive dialysis over periods of several days can also reduce the viability of some phage preparations (see below).

**Fig 2:**
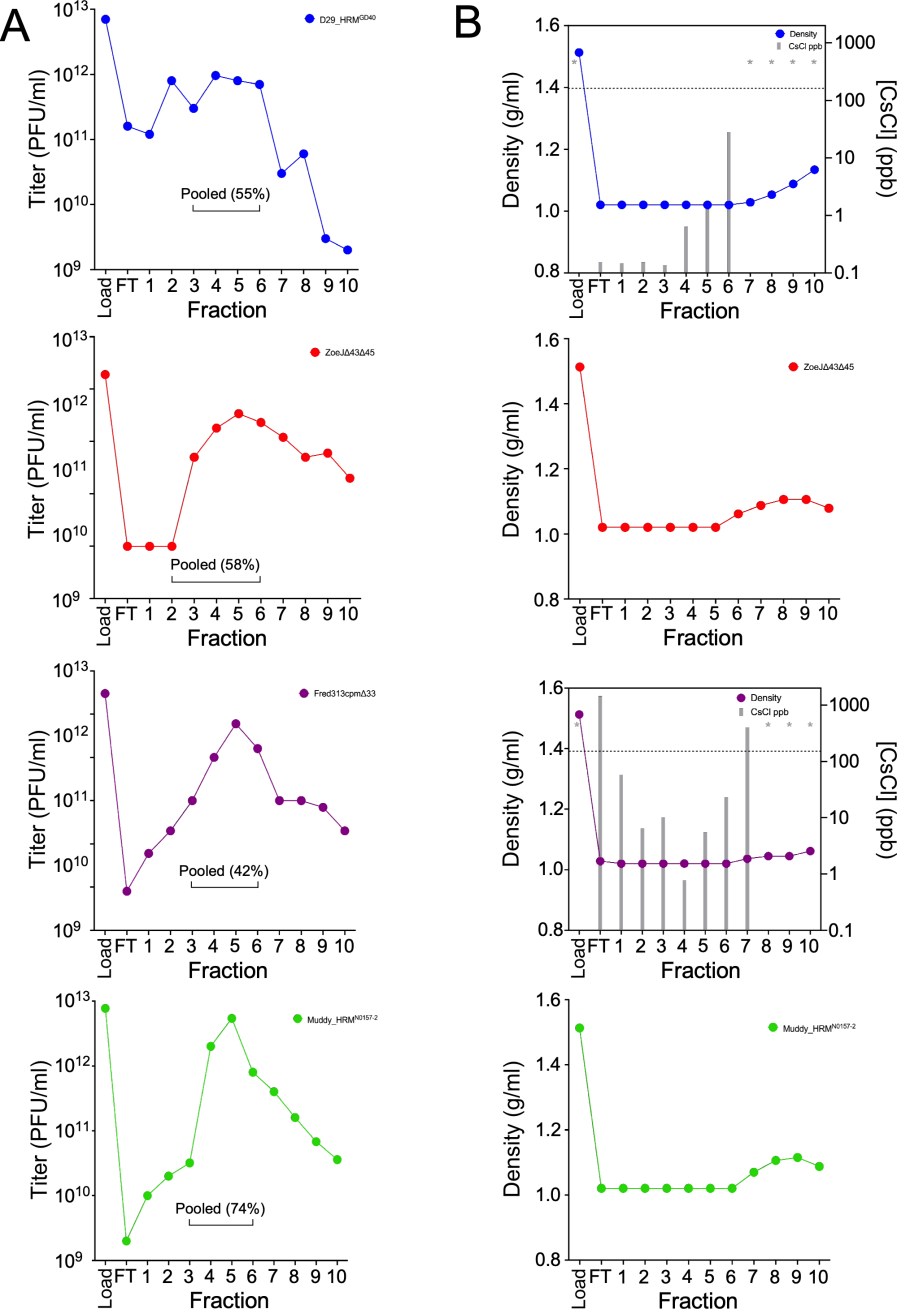
Removal of CsCl using column chromatography. (**A**) Elution profiles of phages through column chromatography. Phage titers (in PFU/mL) of the pre-column material, immediate flow-through, and 1 mL fractions collected during size exclusion chromatography are shown. Sephadex G-100 was used for Fred313cpm∆33, and the others used Sephacryl S-300. Phages D29_HRM^GD40^, ZoeJ∆*43*∆*45*, Fred313cpm∆*33*, and Muddy_HRM^N0157-2^ are shown in blue, red, purple, and green, respectively. The pooled fractions and the yield (percentage of input phage) are indicated. (**B**) Relative density and Cs concentrations of corresponding fractions shown in panel A. Densities are shown as g/mL, and Cs concentrations determined by ICP-MS are shown in parts per billion (ppb) for D29_HRMGD40 and Fred313cpm∆33. Asterisks indicate measurements above the ICP-MS calibration curve. Acceptable levels of Cs in individual fractions were considered to be ~ 100 ppb, which is below the level of 190 ppb (shown by dotted line) reported previously in dialyzed samples (6).

Size-exclusion column chromatography offers an alternative approach to the removal of CsCl from phage preparations, with the potential advantage of being relatively quick and thus perhaps avoiding substantial titer reductions. To explore this, we prepared columns with various resins including Sephadex G-10, G-25, G-100 (Sigma-Millipore), and Sephacryl High-Resolution S-300 and S-500 (Cytiva) and compared them for phage recovery as well as relative density (Fig. S1). Comparisons using similar types of phage preparations showed that Sephadex G-100 and Sephacryl S-300 resins gave the most desirable profiles (Fig. 2; Fig. S1). Using a standardized protocol, different phage preparations had similar profiles in which the immediate flow-through fractions contained relatively low phage concentrations, and maximal phage titers were recovered in fractions #4–6 (Fig. 2; Fig. S1). Some preparations showed higher phage concentrations in the immediate flow-through sample (Fig. 2; Fig. S1), perhaps due to aggregation of the phage particles, a phenomenon described previously (29). Overall, we estimate that fractions 4–6 in total typically include >80% of the input phage sample.

The relative density of the phage preparations added to the resins is ~1.5 g/mL, and we determined the relative densities of each of the fractions collected using a refractometer. The flow-through and early fractions (1–6) typically have densities near 1.0 g/mL (Ringer’s solution has a density of 1.02 g/mL), and small increases in density were observed starting between fractions 5 and 7, although it varies somewhat with different preparations and resins (Fig. 2; Fig. S1 and S2). For three different preparations, we determined the amounts of cesium in various fractions using ICP-MS (Fig. 2B; Fig. S2). The flow-through and early fractions (1–5) typically have <10 ppb cesium, and small amounts of cesium are present in fractions 5 and 6, but less than 100 ppb. Fractions #7 and later have higher amounts of cesium that were not quantifiable as they were outside the calibration range of the instrument (Fig. 2). By pooling fractions 4–6, the total cesium concentration is estimated to be no more than 30 ppb, below that typically measured for dialyzed samples. The total time from loading the column to collection of all fractions is approximately 30 min. Although some differences in phage behaviors were observed, these don’t obviously correlate with any particular features of the phages (Table 1).

**Fig 3:**
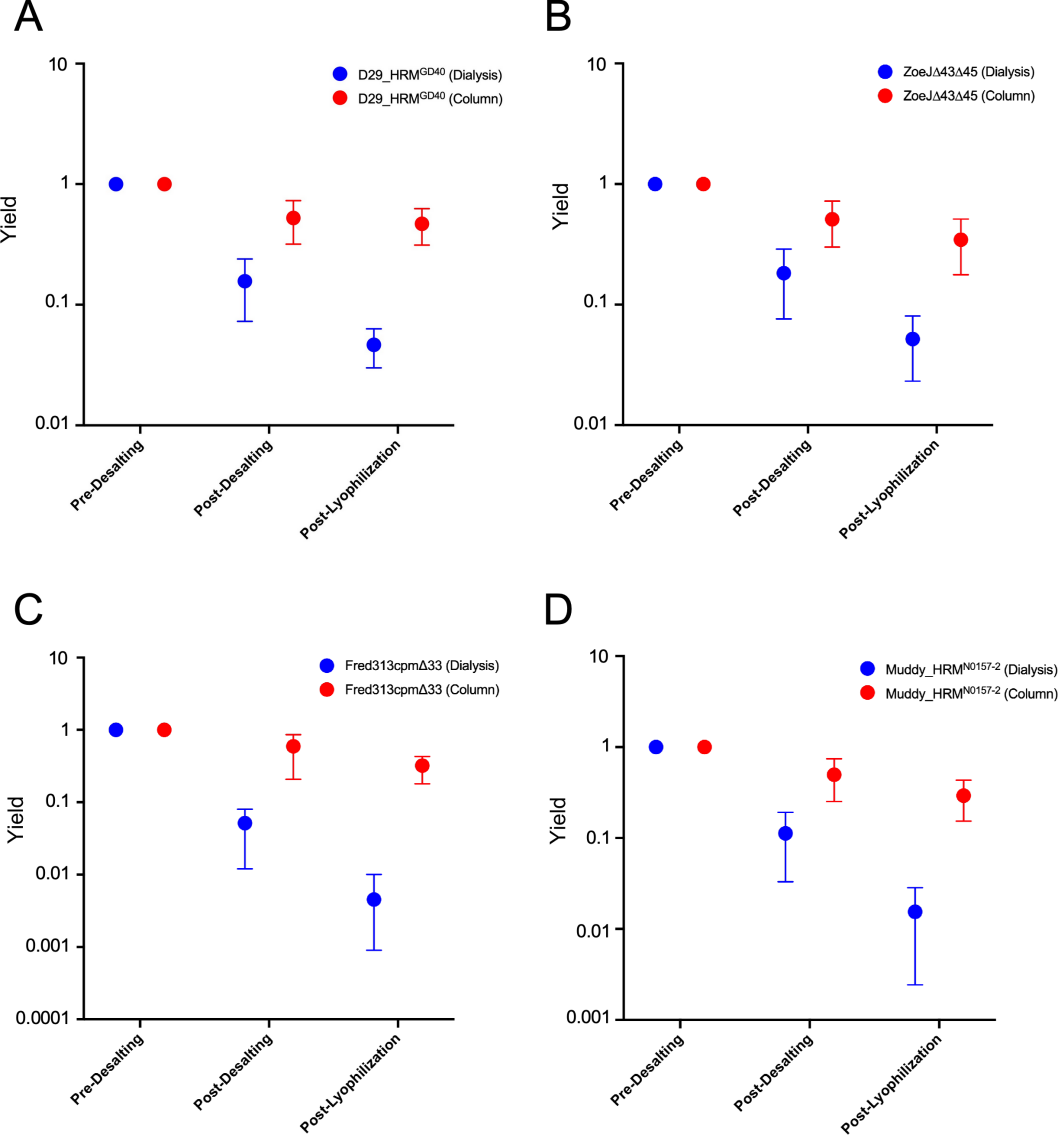
Impact of purification steps on phage titers. (**A–D**) For each phage tested (D29_HRM^GD40^, ZoeJ∆*43*∆*45*, Fred313cpm∆*33*, and Muddy_HRM^N0157-2^ in panels **A–D**, respectively), relative phage titers are shown at each step in the purification. The starting phage titers (pre-desalting) are normalized to 1. The changes in phage yields are shown relative to this after de-salting (either by dialysis or chromatography, shown in blue and red, respectively), and following suspension immediately after lyophilization (post-lyophilization). The yield relative to the starting material is normalized for changes in volume and dilution.

We compared the phage concentrations in several phage preparations depending on whether the CsCl was removed by dialysis or chromatography (Fig. 3). For all the phages tested, decreases in the titer were seen for both dialysis and chromatography, but the dialysis resulted in considerably greater reductions (Fig. 3). For the chromatographic samples, the yield of phage primarily results from selection of just the peak fractions, and viability is not a main cause of loss. For the dialysis samples, reductions in titer are mainly due to reduced viability, which contributes to greater loss than the reduction in yield by chromatography.

### Lyophilization and storage

Following desalting, phage preparations were lyophilized in an attempt to achieve improved conditions for storage and shipping that are not dependent on a cold chain. In preliminary experiments, we observed that lyophilization without any added excipient resulted in a large reduction in viability (>10^6^ fold reduction). Trehalose has been used extensively as an excipient in lyophilization (30), and spray drying of bacteriophages was performed, due to its high glass transition temperature (31), so we explored this further. When including a final concentration of 0.5 M trehalose in Ringer’s solution, we observed good maintenance of viability following lyophilization using a protocol that helps form a suitable dry cake (Fig. 3). Interestingly, when the phage preparations were desalted using dialysis, the viability following lyophilization was substantially lower than when using column chromatography (Fig. 3). These general trends were observed for all four phages tested and were sufficiently reproducible—particularly when using chromatography—that a desirable specific final concentration of viable phage particles could be achieved by adjusting the input phage concentration to compensate for the loss. Typically, we targeted a total viable phage load in each lyophilization vial of 10^10^ PFU, such that reconstitution in 10 mL Ringer’s solution yielded ten 1 mL doses of 10^9^ PFU/dose.

### Stability of lyophilized phage preparations

**Fig 4:**
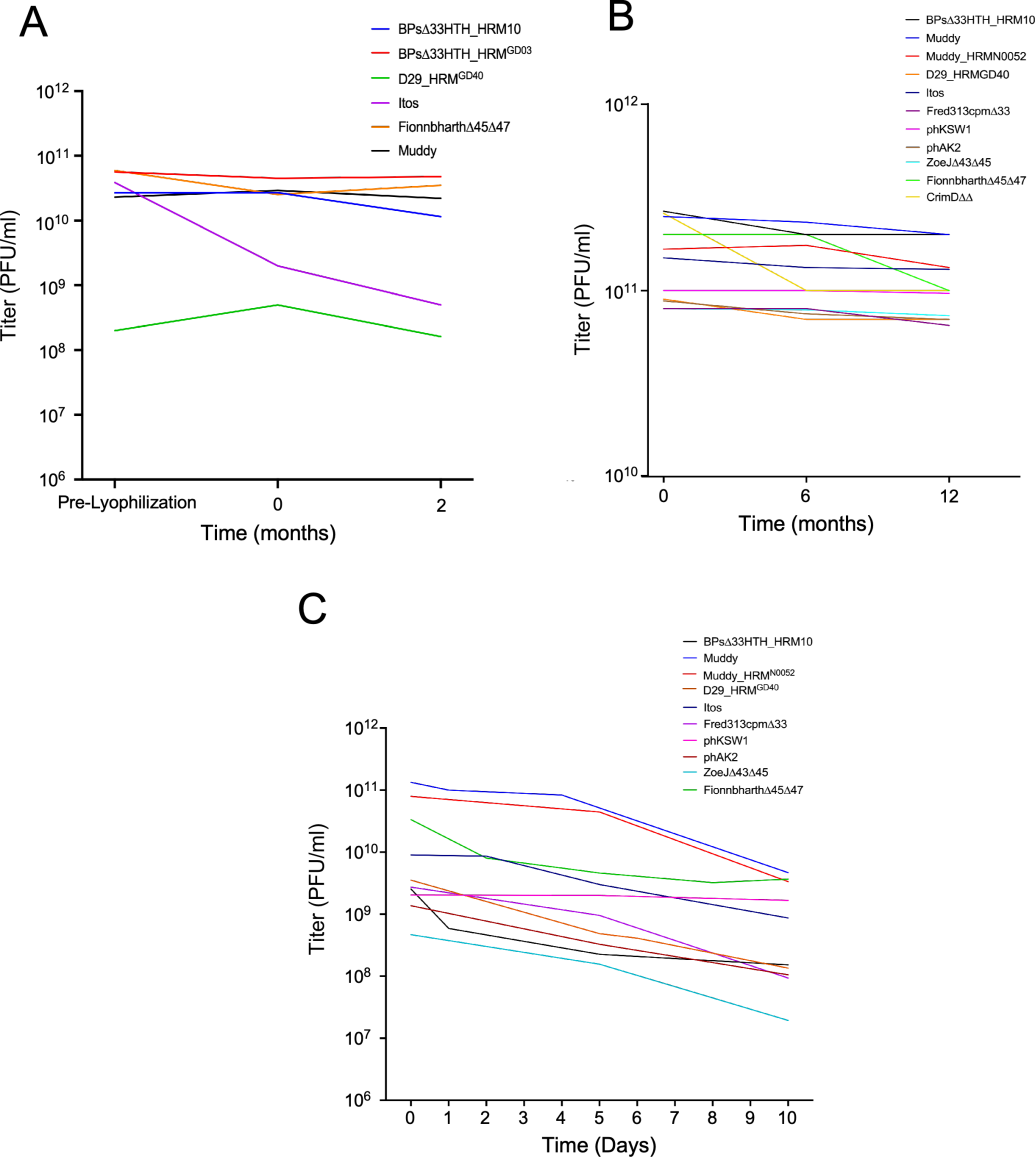
(**A**) Stability of lyophilized bacteriophage samples for 2 months at room temperature. Phage titers are shown for preparation prior to lyophilization, immediately following lyophilization (0 months) and after 2 months at room temperature. (**B**) Stability of lyophilized bacteriophage samples over 12 months at 4°C. At either 6 or 12 months, the contents of a lyophilized vial were resuspended and the titers determined. (**C**) Stability of resuspended lyophilized bacteriophage samples in Ringer’s over 10 days. Contents of lyophilized vials were resuspended in 10 mL Ringer’s solution and distributed to ten 1 mL syringes, stored at 4°C, and the titers determined over the course of 10 days.

To obtain initial stability data, we stored lyophilized phage samples at room temperature for 2 months and determined any changes in the titer (Fig. 4A). Two preparations of BP derivatives, a Fionnbharth derivative, and phage Muddy showed minimal differences in titer between pre-lyophilized, lyophilized without storage, and after 2 months room temperature storage (Fig. 4A). Phage Itos and a D29 derivative showed some reduction in titers after lyophilization and also after 2 months of storage at room temperature (Fig. 4A). We also tested a series of phage preparations after storage at 4°C for up to 12 months (Fig. 4B). In general, the phages showed good stability, although some had seemingly nonlinear responses, and some were more stable than others (Fig. 4B). Even the maximal losses were less than 10-fold over a 12 month period under these conditions (Fig. 4B).

We also determined the stability of the phage preparations after lyophilization, resuspension in Ringer’s solution, distribution into ten 1 mL syringes, and stored at 4 °C (Fig. 4C). After 5 days, the loss in titers was typically less than 10-fold, and most phages did not lose more than about 10-fold titer in 10 days. We have observed that once phages were diluted and stored in syringes, their viability was influenced by air travel, resulting in substantial loss of titers compared to identical samples that did not travel. In contrast, lyophilized samples (a BP derivative) have been shipped about 20,000 miles by air (Pittsburgh to Sydney, Australia, and back), resuspended, titered, and showed little or no reduction in viability.

## DISCUSSION

We have described here methods that are suitable for preparation of mycobacteriophages appropriate for compassionate use therapies and which may provide helpful insights into phage preparation for other uses including clinical trials. None of the component methods are novel, but the application and characterization of the parameters are useful. We note that authorization by the United States Federal Drug Administration (FDA) through a single patient or emergency Investigational New Drug (eIND) application does not typically require phage production using Good Manufacturing Processes (GMP), but the preparations must be certified as sterile and must have tolerable levels of endotoxin contamination. Because *Mycobacterium* strains do not contain lipopolysaccharides, they are not an expected contaminant of mycobacteriophage preparations, although other bacterial components could be potentially toxic or have undesirable adverse reactions. Reduction of contaminating bacterial components is thus an important facet of any purification scheme, particularly when phage preparations will be regulatory delivered intravenously over the course of months or even years.

Purification of phage particles by CsCl equilibrium density ultracentrifugation is a well-established method (32, 33), and it has been noted previously that banding twice in CsCl greatly increases the purity (32). The phage preparations typically maintain viability when stored in CsCl solutions, but we note that this is not true for all phages (33). Many phages, including all those described here, have a relatively density of approximately 1.5 and therefore either sink or float in a density gradient to near the middle of the centrifuge tube. Most other cellular components have different densities than this and therefore band in different parts of the gradient. Ribosomes also have a density close to 1.5, so they could co-purify with phage particles, although we do not see overrepresentation of ribosomal proteins in the LC-MS/MS spectra mapping to host proteins.

A potential concern raised by some regulators is whether unpredictable, and undesired events could occur during therapy if the phage preparation contains generalized transducing particles, i.e., those containing errantly packaged bacterial DNA instead of phage DNA. The phage preparations sequenced here had extremely low levels of contaminating host DNA, in contrast to sequences from a phage that is expected to mediate some level of generalized transduction. In addition, we note that all of the phages reported here with therapeutic potential have *cos*-type DNA packaging systems, and such phages typically do generalized transduction only rarely (34). Furthermore, it is plausible that at least some of the phages actively degrade the host genome during replication, which would diminish the extent of host DNA contamination.

Lyophilization is a well-established method for storing phages, and here we have defined the parameters for phage stability in lyophilized preparations, both for long-term storage (i.e., 12 month at either 4°C), and in syringes when reconstituted. We find that 0.5 M trehalose is an excellent excipient, and we note that many other biological therapeutics approved by the FDA for intravenous use have higher concentrations of trehalose than our reconstituted preparations, which have 1.7 mg of trehalose; for example, HERCEPTIN—trastuzumab and MONJUVI—tafasitamab contain 136.2 and 378.3 mg, respectively. Furthermore, we find that the 1.7 mg/mL of trehalose present in each dose only adds a 5 mOsm/L to Ringer’s approximate 280 mOsm/L, staying in the isotonic range and ensuring IV suitability.

Finally, we show that column chromatography has advantages over dialysis for removal of the CsCl after ultracentrifugation. It is quick, the yield is acceptable, and only modest losses in viability are observed relative to dialysis. Moreover, the chromatography-prepared phage particles appear to maintain viability somewhat better through the process of lyophilization. It efficiently removes the CsCl, and the residual CsCl concentrations are many orders of magnitude lower than doses of CsCl administered as potential cancer therapies.

We anticipate the detailed reporting of these methods will facilitate replication for mycobacteriophages and for other phages that are to be considered for compassionate-use therapies.

## MATERIALS AND METHODS

### Bacteria and phages used in this study

*M. smegmatis* mc^2^155 is a high-efficiency transformation strain that has been reported previously (35). Phages D29_HRM^GD40^ (8), ZoeJΔ*43*Δ*45* (6, 7), Fred313cpmΔ*33* (7), Muddy (6, 7), Muddy_HRM^N0157-2^ (36), BPsΔ*33*HTH_HRM10 (6, 7), FionnbharthΔ*43*Δ*45* (36), Itos (6) and the BPsΔ*33*HTH_HRM10 (6), and its derivative phKSW1 (37) have been reported previously. Phage phSY1 is a derivative of CrimDΔ41-43 (itself a lytic derivative of CrimD with engineered deletions of integrase and repressor genes*—41* and *43*, respectively—as well as gene *42*, which is of unknown function) and is a host range mutant isolated on *Mycobacterium chimaera* GD357. Genome sequencing showed that it has three single-nucleotide substitutions relative to parental CrimD in addition to the engineered deletions. These are at G22948C conferring a G700A substitution in gp27, a predicted minor tail, and is presumably responsible for the change in the host range, and two synonymous changes at C15407T and one at G16162T in genes *21* and *22*, respectively.

### Growth of *M. smegmatis*

Starter cultures of *M. smegmatis* mc^2^155 were prepared by inoculation of a single colony to 3 mL of Middlebrook liquid medium containing Middlebrook 7H9 (BD Difco, 271310) supplemented with glucose (0.2%), calcium chloride (1 mM), and Tween-80 (0.05%) (i.e., 7H9/glucose/Ca/Tw). Cultures were incubated with shaking at 37°C until saturated, and 100 µl was used to inoculate 200 mL of 7H9/glucose/Ca/Tw in a baffled flask. This was grown with shaking at 37°C for about 4 days or until saturated. Although Tween-80 inhibits growth of some mycobacteriophages, *M. smegmatis* hydrolyzes Tween-80 as it grows, and these cultures are suitable for phage propagation.

### Phage amplification

To determine the optimal amount of phage to add for amplification on solid medium, a series of infections were tested for each phage, varying the input from a 10^−1^ dilution of a phage lysate stock to a 10^−6^ dilution. Ten microliters of each phage dilution was used to inoculate 1 mL of a saturated *M. smegmatis* culture and allowed to adsorb for 20 mins at room temperature. Nine milliliters of molten Middlebrook 7H9 (BD Difco) Top Agar (0.35% agar, 1 mM CaCl_2_) was added, mixed, and poured onto solid media, Middlebrook 7H10 (Difco, 262710) in 150-mm-diameter plastic Petri dishes (VWR). After ~48 hours of incubation, plates were flooded with 10 mL phage buffer (68  mM NaCl, 10  mM Tris HCl pH 7.5, 10  mM MgSO_4_, 10  mM CaCl_2_), incubated for 24 hours at 4°C, and the liquid collected. This was centrifuged at 7,000 × *g* for 10 minutes, and the supernatant was collected and filter-sterilized (Thermo Scientific Nelgene Rapid-Flow Sterile Disposable Filter Units, #09-741-02, 75 mm diameter, Sterile, 0.2 um, 500 mL volume, PES membrane material). Phage titers were determined, and the input phage concentration that yielded the highest titer was selected for further amplification.

A standard large-scale phage preparation on solid medium used 40 150-mm-diameter Petri dishes. Using a 500 mL flask, 40 mL of a saturated culture of *M. smegmatis* mc^2^155 (~ 3 x 10^10^ CFU) was mixed with the appropriate volume of phage (as determined above and varying from 1.2 × 10^4^ and 8 × 10^4^ PFU) and allowed to adsorb for 20 mins at room temperature. Four hundred milliliters of molten (55°C) top agar (Middlebrook 7H9 with 1 mM CaCl_2_; “MBTA”) was then added, and 10 mL was poured onto each of 40 plates and allowed to solidify. After a 2-day incubation at 37°C, the top agar layer was scraped from the plates and collected in 250 mL sterile centrifuge tubes. It was then clarified at 7,000 × *g* for 10 minutes (Sorvall Lynx 6000, F12:6 × 500 LEX rotor) at 4°C, and the supernatant containing the phage lysate was collected. Phage titers were typically between 10^10^ and 10^12^ PFU/mL.

The phage preparation was concentrated by centrifugation at 100,000 × *g* for 1 hour at 18°C (Beckman Optima XPN-100 Ultracentrifuge, Ti45 rotor, 29,000 rpm) in 96 mL sealed tubes (Beckman Coulter 345776). The supernatant was removed, and the phage pellets were resuspended in a total volume of ~10 mL of phage buffer; the sample was further clarified by centrifugation at 7,000 × *g* for 10 minutes at 4°C and the supernatant collected. Cesium chloride was added to give a relative density. of 1.5 (~.85 g CsCl per mL lysate) and added to a 14 mL sealed tube (Beckman Coulter 342413) and spun for 16 hours at 132,600 × *g* at 18°C (Beckman Optima XPN-100 Ultracentrifuge, Ti70.1 rotor, 38,000 rpm). A visible phage band in the central part of the tube was removed by lateral insertion of a syringe with an 18-gage needle, targeting collection of approximately 1–2 mL. The volume of this sample was then increased to a total of 14 mL using a solution of phage buffer containing CsCl to a density of 1.5 g/mL, and the ultracentrifugation was repeated. Approximately 1–2 mL was collected by lateral insertion of a needle and syringe, as described above. Phage titers were typically between 10^11^ and 10^14^ PFU/mL.

### Dialysis

One mL of CsCl-banded bacteriophage samples was loaded into a 10K MWCO Slide-A-Lyzer dialysis cassette (Thermo Fisher, 66380) and let to soak in a 2 L sterile glass beaker with 200 mL of Ringer’s solution for approximately 16 hours at 4°C. An additional 400 mL of Ringer’s solution was added to the beaker, soaked for 5 hours, and then another 400 mL was added, and the beaker was placed on a stir plate to be stirred overnight. After 16 hours, the total 1 L of Ringer’s solution was discarded from the beaker and replaced with 1 L of fresh medium. The dialysis cassette was subjected to additional three 1 L buffer exchanges after 4 hours, 2 hours, and 1 hour. The sample was extracted from the cassette and filter sterilized using a 0.22 µm filter. (Genesee Scientific, #25-244, 13 mm diameter, Sterile, PES membrane material). Dialyzed samples were titered on lawns of *M. smegmatis* and stored at 4°C.

### Column chromatography

For purification and removal of CsCl from phage preparations, sterile, disposable polypropylene 60 mL columns with 20 µm polyethylene frits (Marvelgent Biosciences, 11-0282-020) were prepared with various resins including Sephadex G-10, G-25, G-100, or Sephacryl High-Resolution S-300 and S-500 (Cytiva Inc., 17001001, 17003301, 17006001, 17059910, 17061310, respectively). These were initially equilibrated with 20% ethanol and used to create a column bed of approximately 10 mL volume with 26.4 mm diameter. Approximately 30 column volumes (~300 mL) of Ringer’s solution were allowed to run through the column without pumping, until the liquid reached the top of the resin bed. Approximately 1 mL of purified phage particles recovered from the equilibrium density gradient centrifugation was carefully loaded onto the resin, and once this entered the column space, an additional 10 mL of Ringer’s solution was loaded onto the bed. Approximately 1 mL of solution flowing through the column immediately after load was collected (“flow through”), and then 10 1 mL fractions were collected. Densities of the fractions were measured using a refractometer, and the levels of cesium chloride were determined using inductively coupled plasma mass spectrometry (ICP-MS), using a PerkinElmer NexION 300X instrument. The limit of detection (bottom range of the calibration curve) is approximately 2 ppb. Phage titers were determined by plating on lawns of *M. smegmatis*. Peak fractions were pooled and filter-sterilized through a 0.22 µM filter (Genesee Scientific, #25-244, 13 mm diameter, Sterile, PES membrane material) and were stored at 4°C.

### Lyophilization

Following CsCl removal, all subsequent handling procedures were performed in a Class 2, type A2 biosafety cabinet (Thermo Herasafe KS), which was thoroughly disinfected along with all small equipment items prior to each use. First, the input titer of bacteriophages was established such that each glass vial would receive approximately 10^10^ PFU and that subsequent reconstitution would yield 10 1 mL syringes each with approximately 10^9^ PFU. In practice, the titers of some phage preparations were not sufficiently high to achieve this, and as such, we aimed to achieve final concentrations as close to this as possible.

Each phage preparation was diluted to a total volume sufficient to add 100 µL to each of the total number of vials to be filled, using Ringer’s solution, and human-use grade trehalose (Sigma Aldrich, 1.02776.1000) was added to a final concentration of 0.5 M. After carefully mixing, 100 µL aliquots were distributed to 2 mL autoclaved and sterile glass vials (DWK Life Sciences Wheaton, 06-406A), and sterile rubber stoppers (DWK Life Sciences Wheaton, 06-447E) were positioned on top of each vial without pressure to close. Racks of approximately 100–140 vials were then placed in a FreeZone Triad Freeze-Dryer (Labconco Inc., 794001010) using a standard cycle of temperature and evacuation, similar to those described elsewhere (23, 38). This cycle included the following steps: i) a pre-freeze hold at −5°C for 1 hour, ii) cooling to −30°C at a rate of 1°C/min, iii) maintaining −30°C for 80 minutes, iv) evacuation for primary drying to 300 µbar for 1,000 minutes, holding the shelf temperature at −30°C, v) temperature increase from −30°C to 25°C for secondary drying over a total of 550 minutes, vi) holding at 25°C for 360 minutes with pressure maintained at 300 µbar, vii) sealing the vials through a platform lift while still under 300 µbar vacuum, and viii) restoring normal pressure over a period of 30 mins, and a rate of 1.66 µbar per minute. The vials were then removed from the lyophilizer and closed with aluminum caps (Fisher, 224182). Lyophilized vials were stored at 4°C and checked for stability every 6 months. Other physical parameters such as moisture content were not determined but are expected to be similar to previously described methods (38) and by the plasticization curve of trehalose (39). To determine stability at the desired dosages, individual vials were resuspended in 10 mL of Ringer’s solution, distributed into 1 mL syringes, stored at 4°C, and titered over the course of 10 days.

To test for viability, a freeze-dried vial was reconstituted in 100 µL sterile deionized water, serially diluted, and plated on a lawn of *M. smegmatis*. Vials were also sent for USP71 sterility testing by Accugen Inc., and endotoxin levels were determined using an EndoZyme II assay kit from Hyglos GmbH.

### Proteomic analysis of phage preparations

A 1 mL phage sample (~10^11^ PFU) was centrifuged at 13,000 rpm for 30 minutes at 4°C, the supernatant removed, and the phage pellet frozen in a bath of dry ice and ethanol. The pellet was resuspended in 50 µL lysis buffer (5% SDS in 50 mM triethylammonium bicarbonate, TEAB), sonicated to disperse, and centrifuged at 13,000 × *g* for 10 minutes at room temperature. The supernatant was recovered, and the protein concentration was quantified with the micro-BCA assay (Thermo Scientific #23235). Ten µg of protein was digested using the S-trap (ProtiFi, LLC) protocol, reduced with 20 mM dithiothreitol, heated to 95°C for 10 min, cooled to room temperature, and incubated with 40 mM iodoacetamide in the dark for 30 min at room temperature. Samples were centrifuged at 13,000 × *g* for 8 min, acidified with 12% phosphoric acid at 1:10 concentration, and diluted six-fold in binding buffer (90% methanol and 100 mM TEAB at pH 7.1) before being dispensed onto S-trap columns (ProtiFi, LLC). The columns were washed with binding buffer, centrifuged at 4,000 × *g* for 1 minute, and incubated in clean tubes at 47°C in 50 mM TEAB for trypsin digestion at 1:20 enzyme/substrate. Samples were eluted stepwise with 50 mM TEAB, then 0.2% formic acid, and finally 50% acetonitrile with 0.2% formic acid, with centrifugation at 1,000 × *g* for 1 min after each step. Eluants were dried in a speedvac and resuspended in a solution of 3% acetonitrile and 0.1% formic acid to a final concentration of 0.2 µg/µL and then desalted using Pierce Peptide Desalting Spin Columns (Thermo Scientific # 89851).

Mass spectrometry analysis was conducted by the University of Pittsburgh BioMS facility using a Bruker timsTOF Pro2 coupled to a NanoElute 2. Approximately 200 ng of peptides was loaded onto an Aurora Ultimate C18 column (1.7 µm, 25 cm x 75 µm, IonOpticks) and peptides eluted at 300 nL/min over a 60 minute gradient. The timsTOF Pro2 was set to PASEF scan mode and DDA with a scan range of 100–1,700 *m/z* with 10 PASEF ramps, and the TIMS was set to a 100 ms ramp with accumulation time (100% duty cycle) and a ramp rate of 9.43 Hz. Linear precursor repetitions were set to a target intensity of 20,000 and a target threshold of 2,500; active exclusion was set to 0.40 min. Collision energy was set to a base of 1.60 1 /_K0_ [V-s/cm^2^] at 59 eV and 0.60 1/ K_0_ [V-s/cm^2^] at 20 eV. The isolation width was set to 2 *m/z* for <700 *m/z* and 3 *m/z* for >800 *m/z*.

The MS data were analyzed using FragPipe v23 and MSFragger 4.3, searching masses against a database consisting of all possible peptides encoded in the six-frame translation of the phage, plus the annotated coding regions of *M. smegmatis* (GenBank CP102346.1). The phage peptide library was created by using a standalone instance of the NCBI ORFfinder program to identify all possible open reading frames in the phage genomes and then translating these into peptides using the translation tool in the Sequence Manipulation Suite webserver (https://www.bioinformatics.org/sms2/translate.html). MS analysis settings used were as follows: missed cleavages = 2; minimum peptide length = 5; precursor mass tolerance = 20 ppm; fragment mass tolerance = 20 ppm; variable modifications: oxidation / +15.9949 Da, N-terminal acetylation / +41.0106 Da; fixed modifications: carbamidomethylation / +57.02146 Da); and a 5% FDR cutoff for both proteins and peptides. Peptide spectral matches reported in Table 2 were filtered with a false discovery rate (FDR) of 0.3%.

### Genomics analysis of phage preparations

Sequencing libraries were prepared using DNA samples from phage preparations and then sequenced by Illumina (either MiSeq, NextSeq, or NovaSeq). Reads were trimmed using fastp (40) with default parameters except for the following: qualified base quality value of 30, average quality of 30, N base limit of 1 per read, and minimum output read length of 50 bases. Trimmed reads were simultaneously aligned to the complete phage genome and the complete genome of the host on which it was propagated using the “Map Reads to Reference” tool in CLC Genomics Workbench (version 23.0.3), with cutoff values of 50% coverage and 80% identity for each read. Due to the relatively small number of reads that aligned to the host genomes, each of those alignments was manually inspected, and any determined to be spurious was discarded from final counts.

## SUPPLEMENTAL MATERIAL

Supplemental Material spectrum.02277-25-s0001.pdf Tables S1 to S3; Fig. S1 and S2.

ASM does not own the copyrights to Supplemental Material that may be linked to, or accessed through, an article. The authors have granted ASM a non-exclusive, world-wide license to publish the Supplemental Material files. Please contact the corresponding author directly for reuse.
