# Supplementary material for: Amplification, purification, and lyophilization of mycobacteriophages for therapeutic use
Source: Microbiol Spectr. 2025 Nov 3;13(12):e02277-25. doi: 10.1128/spectrum.02277-25 (PMC12671170; doi:10.1128/spectrum.02277-25)
Supplement: Supplemental Material — Tables S1 to S3; Fig. S1 and S2. [file spectrum.02277-25-s0001.pdf]

Supplementary Materials

Table S1. ICP-MS determination of Cs concentrations in purification of D29_HRM^{GD40} lot#146

Fraction	Cs (ppb)	Density (g/ml)	Titer (PFU/mL)
Pre-Column	S	1.513	9×10^{11}
Flow-Through	2240	1.0284	$<5 \times 10^{7}$
Fraction #1	0.169	1.0204	$<5 \times 10^{7}$
Fraction #2	0.01	1.0204	$<5 \times 10^{7}$
Fraction #3	0.135	1.0204	1×10^{9}
Fraction #4	11.8	1.0204	4.5×10^{11}
Fraction #5	5.48	1.0204	3.2×10^{11}
Fraction #6	87.8	1.0204	3.50×10^{10}
Fraction #7	S	1.0284	5.0×10^{9}
Fraction #8	S	1.1245	1×10^{9}
Fraction #9	S	1.2387	5.5×10^{8}
Fraction #10	S	1.1151	6.5×10^{8}

Table S2. ICP-MS determination of Cs concentrations in purification of D29_HRM^{GD40} lot#159

Fraction	Cs (ppb)	Density (g/ml)	Titer (PFU/mL)
Pre-Column	S	1.513	4×10^{12}
Flow-Through	0.156	1.0204	1×10^{9}
Fraction #1	0.146	1.0204	2×10^{9}
Fraction #2	0.155	1.0204	1.5×10^{10}
Fraction #3	0.137	1.0204	1×10^{12}
Fraction #4	0.634	1.0204	9×10^{11}
Fraction #5	1.42	1.0204	8×10^{11}
Fraction #6	28.1	1.0204	7×10^{10}
Fraction #7	S	1.0365	4.7×10^{8}
Fraction #8	S	1.0615	2.5×10^{8}
Fraction #9	S	1.0877	1×10^{9}
Fraction #10	S	1.07	7×10^{8}

Table S3. ICP-MS determination of Cs concentrations in purification of Fred313cpmΔ33.

Fraction	Cs (ppb)	Density (g/ml)	Titer (PFU/mL)
Pre-Column	S	1.513	3.3×10^{12}
Flow-Through	1450	1.0284	5.5×10^{8}
Fraction #1	58.3	1.0204	8×10^{10}
Fraction #2	6.38	1.0204	1.4×10^{11}
Fraction #3	10.02	1.0204	5×10^{11}
Fraction #4	0.767	1.0204	8×10^{11}
Fraction #5	5.54	1.0204	2×10^{11}
Fraction #6	25	1.0204	6.5×10^{10}
Fraction #7	4040	1.0365	5.6×10^{10}
Fraction #8	S	1.0447	2×10^{9}
Fraction #9	S	1.0447	6.5×10^{8}
Fraction #10	S	1.0615	5×10^{8}

Figure S1. A. **Comparison of different resins for phage purification and de-salting**. **A and B**. Phage of D29_HRM^{GD40} preparations were loaded onto various resins and the titers of the pre-column, immediate flow through and subsequent 1 ml fractions are shown (A), with the relative densities shown in panel B. Resin used are Sephadex™ G-10, Sephadex™ G-25, Sephadex™ G-100, Sephacryl™ S-300, and Sephacryl™ S-500, shown in blue, red, green, purple, orange, respectively. **C and D**, Purification of ZoeJΔ*43*Δ*45*, **E and F**. Purification of Fred313cpmΔ*33*, **G and H**. purification of Muffy_HRM$^{N0157\text{-}2}$, all labeled similarly.

Figure S2. Additional de-salting example using column chromatography.

A. Elution profiles of phages through column chromatography. Phage titers (in PFU/mL) of the pre-column material, immediate flow-through, and 1 mL fractions collected during size exclusion chromatography are shown. Sephadex G-100 was used for Fred313cpmΔ33, and the others used Sephacryl S-300. Phages D29_HRM^{GD40}, ZoeJΔ*43*Δ*45*, Fred313cpmΔ*33*, and Muddy_HRM$^{N0157\text{-}2}$ are shown in blue, red, purple and green, respectively. The pooled fractions and the yield (percentage of input phage) are indicated. Relative density of corresponding fractions shown in g/ml.

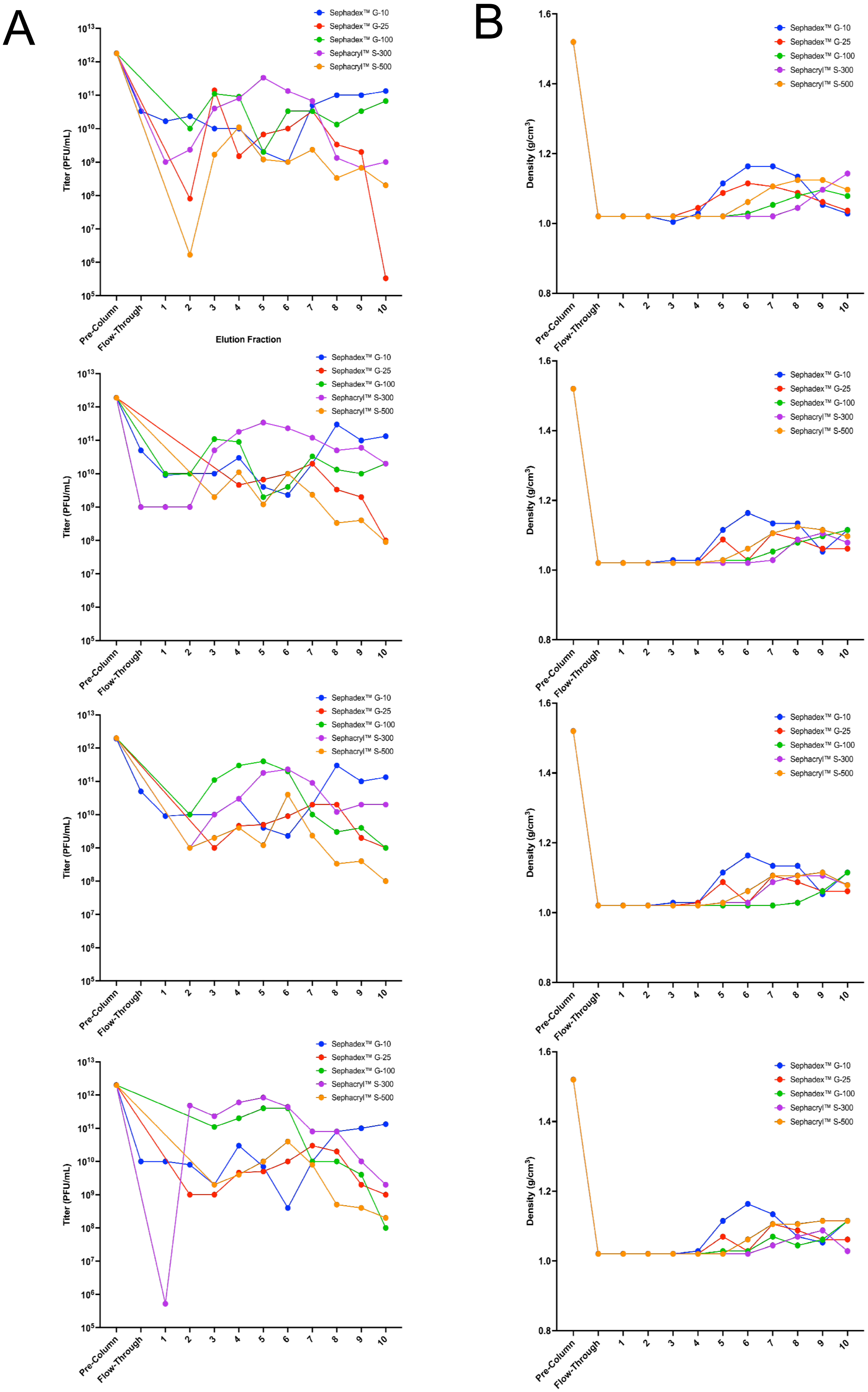
A
B
Sephadex™ G-10
Sephadex™ G-25
Sephadex™ G-100
Sephacryl™ S-300
Sephacryl™ S-500
Titer (PFU/mL)
Density (g/cm³)
Elution Fraction
Pre-Column
Flow-Through
1
2
3
4
5
6
7
8
9
10
10^{5}
10^{6}
10^{7}
10^{8}
10^{9}
10^{10}
10^{11}
10^{12}
10^{13}
0.8
1.0
1.2
1.4
1.6

Figure S1

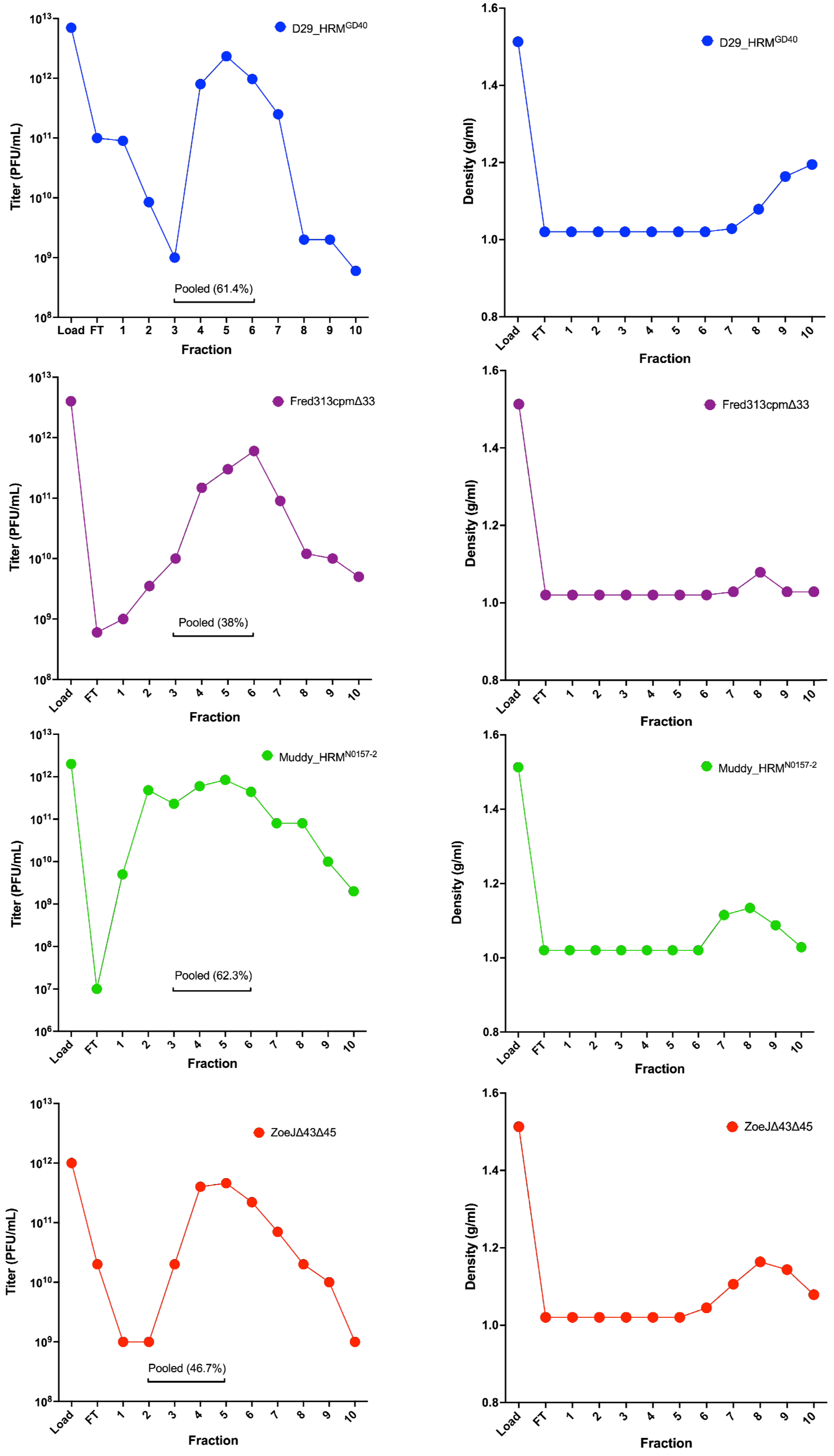

D29_HRM^GD40
Fred313cpmΔ33
Muddy_HRM^N0157-2
ZoeJΔ43Δ45
Titer (PFU/mL)
Density (g/ml)
Fraction
Load
FT
Pooled (61.4%)
Pooled (38%)
Pooled (62.3%)
Pooled (46.7%)

Figure S2
